# Arthroscopic-assisted reduction and internal fixation for complex tibial plateau fracture: radiographic and clinical outcomes with 2- to 15-year follow-up

**DOI:** 10.1186/s13018-023-03938-8

**Published:** 2023-06-22

**Authors:** You-Hung Cheng, Cheng-Pang Yang, Shih-Sheng Chang, Chun-Jui Weng, Chih-Hao Chiu, Yi-Sheng Chan

**Affiliations:** 1 Department of Orthopedic Surgery, New Taipei Municipal Tu-Cheng Hospital, Chang Gung Memorial Hospital, New Taipei City, Taiwan, ROC; 2 Department of Orthopedic Surgery, Linkou Chang Gung Memorial Hospital, Taoyüan, Taiwan, ROC; 3 Present Address: Department of Orthopedic Surgery, Chang Gung Memorial Hospital, Keelung Branch, No.222, Maijin Rd., Anle Dist., Keelung City, 204 Taiwan, ROC; 4 Comprehensive Sports Medicine Center, Linkou Chang Gung Memorial Hospital, Taoyüan, Taiwan, ROC

**Keywords:** Complex tibial plateau fracture, Arthroscopic-assisted reduction, Minimally invasive internal fixation

## Abstract

**Background:**

To investigate the radiologic and prognostic outcomes after using arthroscopic-assisted reduction and internal fixation (ARIF) in complex tibial plateau fractures with mid- to long-term follow-up.

**Methods:**

This retrospective study reviewed complex tibial plateau fractures that underwent ARIF from 1999 to 2019. Radiologic outcomes, including tibial plateau angle (TPA), posterior slope angle (PSA), Kellgren–Lawrence classification and Rasmussen radiologic assessment, were measured and evaluated. The prognosis and complications were assessed by the Rasmussen clinical assessment with a minimum follow-up of 2 years.

**Results:**

Ninety-two consecutive patients (mean age: 46.9 years) with a mean follow-up of 74.8 months (24–180) were included in our series. Using AO classification, there were 20 type C1 fractures, 21 type C2 fractures, and 51 type C3 fractures. All the fractures achieved solid union. TPA was maintained well on average at the last follow-up and showed no significant difference compared to postoperatively (*p* = 0.208). In the sagittal plane, the mean PSA increased from 9.3 ± 2.9° to 9.6 ± 3.1° (*p* = 0.092). A statistically significant increase in PSA was also noted in the C3 group (*p* = 0.044). Superficial or deep infection was noted in 4 cases (4.3%), and total knee arthroplasty (TKA) was performed in 2 cases (2.2%) due to grade 4 osteoarthritis (OA). Ninety (97.8%) and 89 (96.7%) patients had good or excellent results in the Rasmussen radiologic assessment and Rasmussen clinical assessment, respectively.

**Conclusions:**

The complex tibial plateau fracture could be treated successfully using arthroscopy-assisted reduction and internal fixation. Most patients achieve excellent and good clinical outcomes with low complication rates. In our experience, a higher incidence of increased slope was noted, especially in type C3 fractures. Reduction of the posterior fragment should be done cautiously during the operation.

**Levels of evidence:**

Level III.

## Background

The treatment of complex tibial plateau fractures remains challenging during daily clinical practice. The definition of these types of fractures can be classified by Schatzker classification as type V or VI or by AO classification as AO/OTA 41-C. Complex tibial plateau fractures account for up to 39% of all tibial plateau fractures and are often accompanied by severe soft tissue injuries or multiple trauma due to the mechanism of high energy [1]. Due to the complicated situation, the rate of complications during or after the operation for complex tibial plateau fractures was reported to be higher than that for simple types. Posttraumatic osteoarthritis (OA) also occurs more frequently due to the complexity of joint surface depression, and the risk for arthroplasty in the long-term follow-up is higher [2–6].

A number of operative procedures have been described for the treatment of complex tibial plateau fractures, and controversy remains regarding the best surgical method [7]. Techniques of arthroscopic-assisted reduction and internal fixation (ARIF) were introduced and have been reported for years with good preliminary results [8–18]. The beneficial outcome can be attributed to direct visualization and reduction of the joint surface, combined treatment of intra-articular soft tissue injury, and a minimally invasive incision compared to traditional open reduction using arthroscopy. However, there remains a paucity of studies focusing on complex tibial plateau fractures using ARIF. The long-term outcomes of fractures in these types are also scarce. The purpose of this study was to investigate the clinical and radiographic prognosis of complex tibial plateau fractures using ARIF in our institute with long-term follow-up.

## Methods

### Study design

**Fig. 1:**
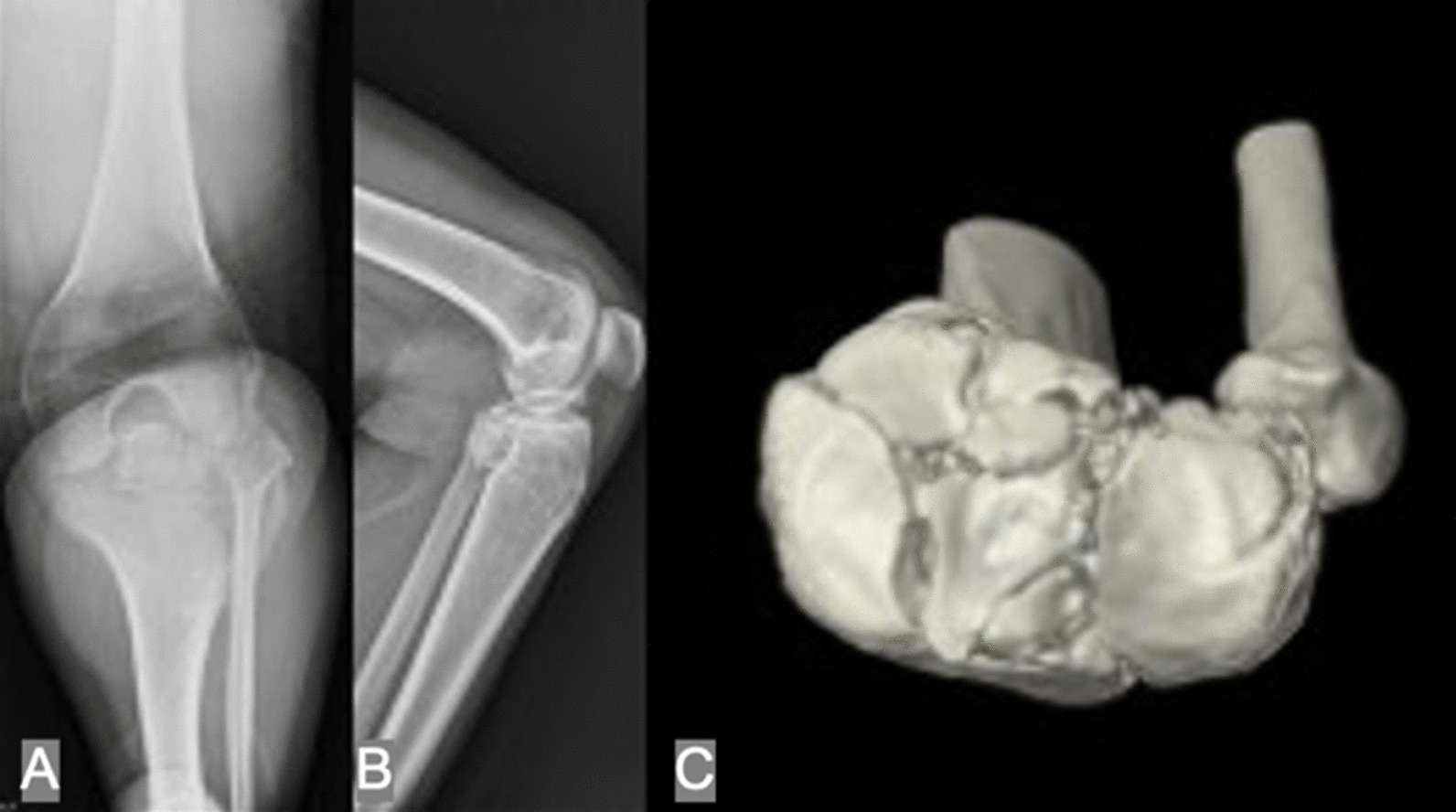
**A** 42-year-old female diagnosed with AO/OTA 41C3 tibial plateau fracture. **A**, **B** Multiple fragments were noticed with bicondylar involvement. **C** Avulsion fracture of the anterior cruciate ligament was also combined with complex tibial plateau fracture

This study was a retrospective cohort review from November 1999 to April 2019 under a protocol approved by the institutional review board. A total of 321 consecutive tibial plateau fractures were treated using arthroscopy-assisted surgery with internal fixation at our hospital. The inclusion criteria in this study included the presence of complex tibial plateau fracture (AO/OTA 41-C) in skeletally mature patients. Patients with pathological fractures, skeletal immaturity, previous surgery, systemic joint diseases, fractures involving the same extremity, prior limitations of motion over knee joint and cases with less than 2 years of follow-up postoperatively were excluded from the study. All the fractures were classified and confirmed by computed tomography (CT) scan before the surgery (Fig. 1).

**Fig. 2:**
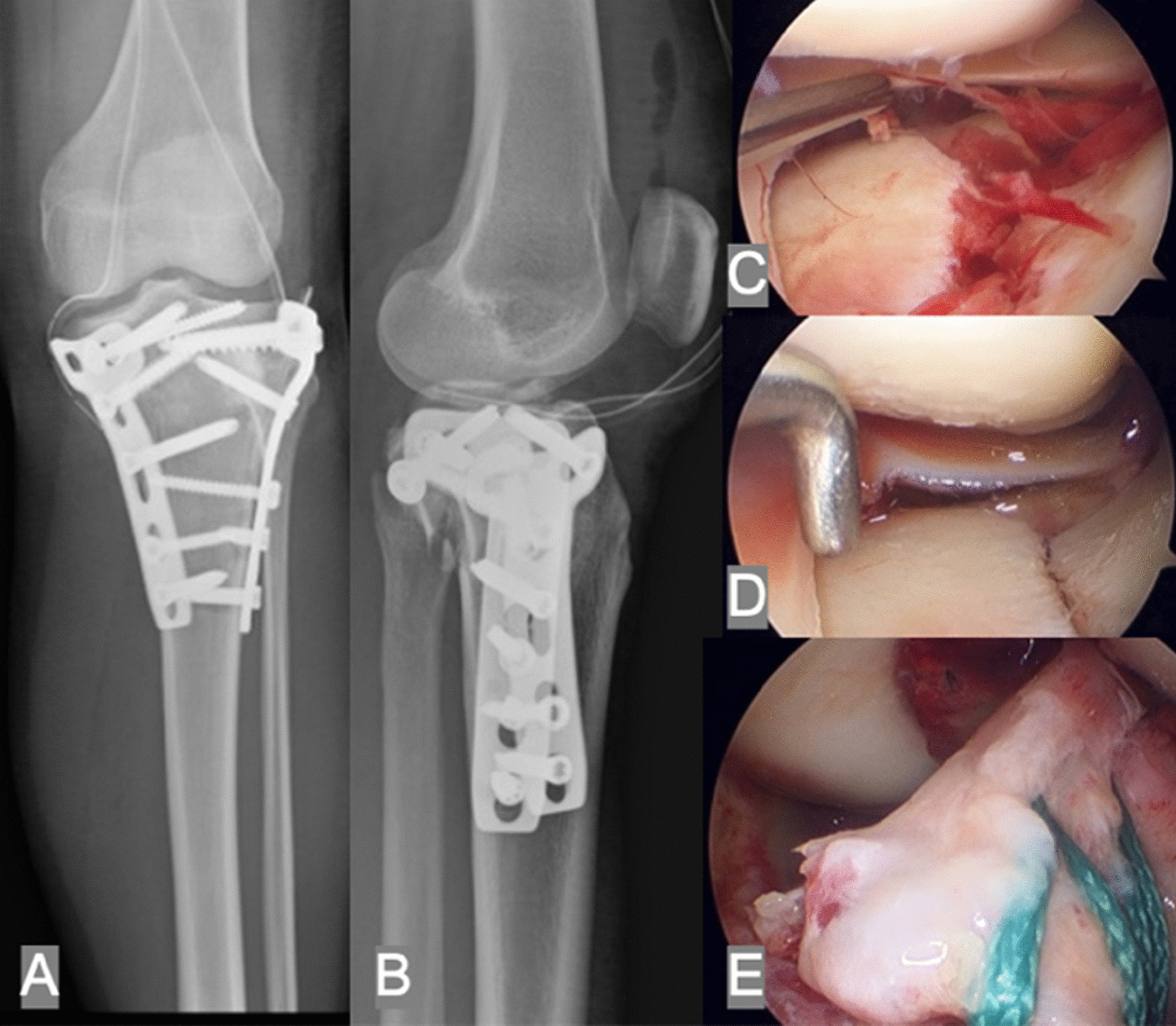
Arthroscopic-assisted reduction and internal fixation intraoperatively. **A**, **B** In addition to double plating, the posteromedial fragment was fixed with 2 interfragment screws. Both planes of radiographic alignment were well maintained. **C**, **D**, **E** Using the arthroscopic technique, joint depression due to the posteromedial fragment was directly visible and could be reduced perfectly through the fracture site. ACL avulsion was also treated simultaneously with arthroscopic suture fixation using 4 No. 5 Ethibond sutures

Patient demographic data, including sex, age and associated injuries, were reviewed and collected from the charts. All included cases were divided into 3 subgroups according to AO/OTA 41 types C1–C3. Radiographic data, including tibial plateau angle (TPA) and posterior slope angle (PSA), were used for assessment of joint alignment on the X-ray postoperatively and at the time of final follow-up (Figs. 2. and 3). Grading of OA was recorded by classification of Kellgren–Lawrence on the X-ray preoperatively and the last follow-up. If the patient had converted to arthroplasty, the last radiograph of the status with internal fixation was used for measurement. The status of union was evaluated using the presence of bridging callus on both planes of radiography. Rasmussen radiologic assessment was used for further evaluation of the quality of reduction on the last radiography. Patient functional outcomes were assessed by the Rasmussen clinical assessment using questionnaires in the clinic. The scores of both assessments were graded as poor, fair, good, and excellent. All patients were examined by an independent observer (Y-H.C.)

**Fig. 3:**
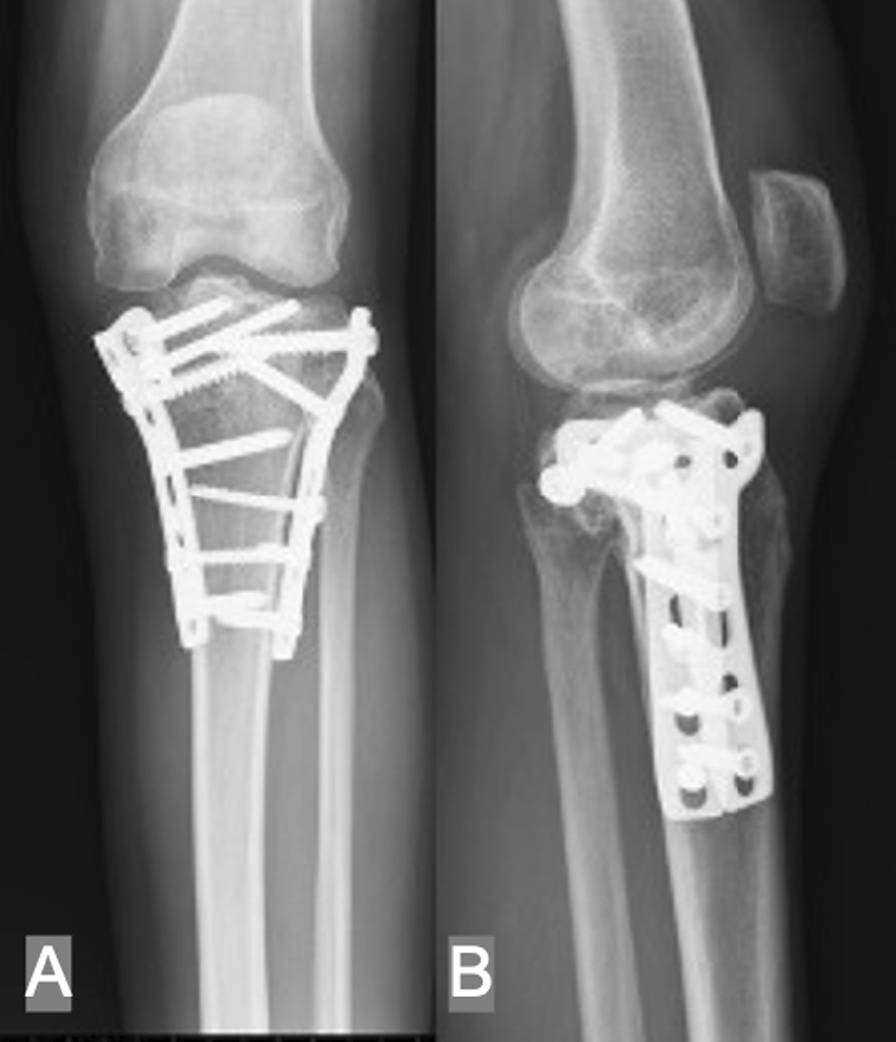
Radiography of the same patient mentioned above 3 years postoperatively. **A**, **B** Both planes of radiographic alignment were well maintained

### Surgical technique

All surgical procedures were performed by a single surgeon (Y-S.C.). Under general anesthesia, patients were in the supine position with a tourniquet over the proximal thigh. Arthroscopic examination was routinely performed first. Standard anteromedial and anterolateral portals were created, and gravity inflow was used to minimize fluid extravasation. In addition to the articular depression, other possible associated intra-articular lesions were inspected and checked meticulously using arthroscopic techniques to further determine the optimal treatment. Hematoma, loose bodies or synovitis were debrided or removed. The associated injuries were treated prior to or following internal fixation according to the type of injury. We preferred to perform meniscal repair or partial meniscectomy prior to fracture fixation due to better accessibility to the meniscus. After cautious evaluation of the soft-tissue structures inside the knee, reduction with a minimally invasive wound was performed. To avoid arthrotomy and extensive soft-tissue stripping, double incisions were performed around the proximal tibia with anteromedial and anterolateral approaches. Two wounds should be separated as far as possible to prevent necrosis of the skin. A cannulated impactor or bone tamp was used through the fracture site over the metaphysis to elevate the fragments (Fig. 2). If there was no space for the impactor to enter, we preferred using a standard anterior cruciate ligament (ACL) tibial guide to alternatively create a window around the proximal tibia. Using the ACL guide, a Kirschner wire could pass through the fracture site precisely. Subchondral bone could be elevated, and the articular surface could be reduced accurately with visualization under the arthroscope. Other techniques for more severe and comminuted tibial plateau fractures around the posteromedial corner were often used, as mentioned in our previous study [19]. After acceptable reduction was performed and confirmed by arthroscopy and an image intensifier, multiple Kirschner wires were used temporarily to maintain the reduction. Interfragment screws were sometimes used for fixation of larger bony fragments. Double plating was then applied for the final fixation. After fixation, valgus and varus stress tests were routinely performed to confirm stability and rule out other ligamentous injuries. Wounds were then closed layer by layer with two drains left in a stable knee. If there were associated ligamentous injuries or avulsion fractures, repair of the associated injuries was performed simultaneously in the acute phase. If the injuries met the criteria for reconstruction, a staged operation would be planned.

### Statistical analysis

Statistical data were analyzed utilizing SPSS software (IBM Corp. in Armonk, NY.). Radiographic data for maintenance of the reduction were compared and evaluated from the postoperative condition to the final follow-up. Data for intergroup comparisons were analyzed by independent *t* tests. Comparisons for intragroup classification by AO/OTA 41 types C1–C3 were performed using one-way analysis of variance (ANOVA). A *p* value < 0.05 indicated statistical significance.

## Results

### General data

A total of 105 patients with a diagnosis of complex tibial plateau fracture were treated with ARIF in our institution. Fourteen patients were excluded with a loss to follow-up rate of 12.5%. Among the final 91 cases, the mean age at surgery was 46.9 years (21–81), and the mean duration of follow-up was 74.8 months (24 to 180). The mean time interval from injury to surgery was 7.8 ± 2.4 days. The periods of follow-up of more than 10 years, 5 years and 2 years were 24 (26.1%), 28 (30.4%) and 40 (43.5%), respectively. Classified by AO/OTA 41, there were 20 type C1 fractures, 21 type C2 fractures, and 51 type C3 fractures. The average duration of the arthroscopic portion of the surgery was 35.7 ± 6.3 min. The main general data of the involved patients and their associated injuries sorted by classification are shown in Table 1.

**Table 1:** General data of complex tibial plateau fractures classified with AO/OTA 41-C and associated soft-tissue injuries

	C1	C2	C3
Number	20	21	51
Age	47.7	47.1	47.4
Sex (Male/Female)	8/12	7/14	28/23
Open fracture	1	1	3
Soft tissue compromise	0	6	7
ACL/PCL tear	1	0	6
ACL/PCL avulsion	4	13	21
Meniscus injury	6	12	27
Other ligament injury	5	3	8

*ACL* Anterior cruciate ligament; *PCL* Posterior cruciate ligament

### Radiologic assessment

At the last follow-up, all the patients achieved bony union under radiography. The mean TPA and PSA were 87.1 ± 2.9° and 9.3 ± 2.9° on the day after surgery, respectively. The reduction remained stable at the final follow-up, with a TPA of 86.7 ± 4.5° (*p* = 0.208) and PSA of 9.6 ± 3.1° (*p* = 0.092). However, the posterior slope revealed a statistically significant difference in the type C3 group (*p* = 0.044). No significant differences in TPA or PSA were noted between the groups (Table 2). Using the Rasmussen radiologic assessment to evaluate the quality of reduction, the mean score was 16.41. Thirty-nine of 92 (42.4%) cases were classified as excellent, and 51 cases (55.4%) were classified as good. Two cases in the C3 group were graded as fair. The mean score of type C1 was statistically superior to that of type C3 (*p* = 0.029) (Table 3). The condition of OA according to Kellgren–Lawrence classification was listed in Table 4. Only two patients revealed grade 4 OA in the last follow-up.

**Table 2:** Results of radiological measurement

	All	C1	C2	C3
Post-operative TPA (°)	87.1 ± 2.9°	87.9 ± 2.2°	87.7 ± 2°	86.5 ± 3.3°
The last follow-up TPA (°)	86.7 ± 4.5°	87.6 ± 3.2°	87.7 ± 3.4°	85.9 ± 5.1°
*p* value	0.208	0.691	0.971	0.174
Post-operative PSA (°)	9.3 ± 2.9°	8.6 ± 2°	9.4 ± 3.8°	9.5 ± 2.8°
The last follow-up PSA (°)	9.6 ± 3.1°	9.1 ± 2.6°	9.6 ± 3.9°	9.8 ± 2.9°
*p* value	0.092	0.539	0.143	0.044*

^*^ A *p*-value of < 0.05 indicated significant difference

*TPA* Tibial plateau angle, *PSA* Posterior slope angle

**Table 3:** Results of Rasmussen radiologic assessment

Fracture type	No. of patients	Mean radiologic score (range)	Excellent	Good	Fair	Poor
All	92	16.41 (10–18)	39	51	2	0
C1	20	17.10 (16–18)	11	9	0	0
C2	21	16.57 (14–18)	8	13	0	0
C3	51	16.08 (10–18)	20	29	2	0

Analysis of Variance (ANOVA): C1–C2: 0.493, C1–C3: 0.029*, C2–C3: 0.411

*A *p*-value of < 0.05 indicated significant difference

**Table 4:** Evaluation of OA pre-operatively and the last follow-up

	Pre-operative K–L classification					The last follow-up K–L classification				
Grade	0	1	2	3	4	0	1	2	3	4
All	73	15	4	0	0	43	28	15	4	2
C1	19	1	0	0	0	13	5	2	0	0
C2	16	4	1	0	0	9	6	5	1	0
C3	38	10	3	0	0	21	17	8	3	2

*OA* Osteoarthritis, *K–L* Kellgren–Lawrence

### Clinical assessment and complications

The mean Rasmussen clinical score was 27.55. Sixty-seven patients (72.8%) were classified as excellent, and 22 patients (23.9%) were classified as good. Three patients were graded as fair. One of the cases was a 52-year-old male with a type AO-OTA 41/C3 fracture combined with medial collateral ligament (MCL) and lateral collateral ligament (LCL) tears. ARIF and ligament repair were performed simultaneously. However, malalignment with valgus deformity was gradually revealed in our clinical follow-up. The other two cases with fair outcomes received arthroplasty due to pain and wounds, as mentioned below in detail. There were no significant differences between the groups (Table 5).

**Table 5:** Results of the Rasmussen clinical assessment

Fracture type	No. of patients	Mean clinical score (range)	Excellent	Good	Fair	Poor
All	92	27.55 (16–30)	67	22	3	0
C1	20	28.05 (27–30)	17	3	0	0
C2	21	27.67 (25–30)	18	3	0	0
C3	51	27.31 (16–30)	32	16	3	0

*p* value of Analysis of Variance (ANOVA): C1–C2: 0.862, C1–C3: 0.466, C2–C3: 0.833

Complications were noted in 6 patients. None of them were directly associated with arthroscopic procedures. Each case of type C2 and C3 fractures developed wound dehiscence with superficial infections noted in our clinic. Symptoms were relieved well after oral antibiotics and cautious wound care, and no further surgery was needed. High energy-related soft tissue compromise might be the reason for the delayed healing of the wound. Deep wound infections were noted in two cases (1 type C1, 1 type C3) with initial open fractures. Debridement was performed, and then admission for antibiotic treatment was arranged for 2 weeks and then shifted to oral form for an additional 4 weeks in both cases. Two patients with comminuted C3 fractures developed grade 4 OA after ARIF for 3 years and 5 years. Conversion to total knee arthroplasty (TKA) was then arranged with satisfactory outcomes.

## Discussion

Until now, there has been no general agreement on how complex tibial plateau injuries should be managed. This is the first study to assess the use of ARIF for the treatment of complex tibial plateau fractures with long-term follow-up. Our series revealed good or excellent long-term prognosis in most of the patients.

The treatment of complex tibial plateau fractures is challenging. Substantial soft-tissue injuries were common, and up to 43% were open fractures [1, 20–22]. Poor soft tissue conditions and comminuted intra-articular fragments related to high-energy mechanisms are the main factors predisposing patients to high complication rates using traditional open reduction and internal fixation (ORIF). Arthroscopic-assisted reduction and internal fixation for tibial plateau fractures was first introduced by Caspari et al. and Jennings, with series focusing on relatively simple fracture types (Schatzker types I, II and III) [17, 18]. Through the years, some techniques have been developed to overcome the more complex plateau fractures under arthroscopy, especially fragments involving the posterior compartment [9, 16, 19, 23]. Several studies have already shown satisfactory functional and radiological results. The incidence of associated injuries, including chondral injury, meniscus tears or rupture of ligaments, was high, followed by tibial plateau fractures. In a large matched cohort study with 7950 cases, 7.6% of tibial plateau fractures needed secondary knee arthroscopy compared to 2.0% of the reference group [24]. In our series, the incidence of associated injury was 69.5% after complex tibial plateau fracture. Although it is more technically demanding, ARIF allows surgeons to treat plateau fractures and intra-articular soft tissue simultaneously. The avoidance of arthrotomy when using arthroscopy could prevent the risk of stiffness, further tenderness or iatrogenic meniscal injuries [25, 26]. Moreover, with the direct visualization of the joint surface, reduction could be made easily with a minimally invasive incision of the wound for internal fixation. The abovementioned benefits might explain the good prognosis in our series.

Reduction of the joint surface is one of the primary goals while treating tibial plateau fractures. In the cases classified by Schatzker type V/VI and AO 41-C, the fracture is usually more comminuted and often involves the posterior compartment. In our experience, using an ACL tibial guide to create an accurate cortical window could efficiently reduce the depressed fragment. Combined with buttress plates and cannulated screws for fixation with arthroscopic techniques, the posterior compartment of the fractures could be restored with well-documented radiographic healing, good clinical outcomes, and low complication rates [19]. We used the same technique for the treatment of complex tibial plateau fractures and used TPA and PSA to evaluate the quality of coronal and sagittal reduction. We noticed no difference in the TPA between the postoperative day and the last follow-up in the AO-41 C1, C2 and C3 groups, which indicated that the reduction in the coronal plane was well maintained. However, increased PSA was shown in the final follow-up in all the groups, with a significant difference in the type C3 group compared to the postoperative radiograph. It revealed the difficult maintenance of the posterior fragment, especially in AO-41 C3 type. Recently, the role of the posterior slope has received more attention due to its influence on stability, activity, and load sharing biomechanically [27–31]. Streubel et al. demonstrated that most patients had significant sagittal displacement that would require surgical reduction of at least one condyle in C-type bicondylar tibial plateau fractures [32]. Barei et al. [20] reported the radiographic result of complex AO/OTA 41-C3 tibial plateau fractures after double plating using ORIF and found that only 72% satisfactory sagittal alignment can be achieved with the range of PSA of 9 ± 5°. Eggli et al. [33] demonstrated that in 13 out of 14 complex tibial plateau fractures, the posterior proximal tibial angle was measured as anatomically aligned. In our series, 87 of 92 (94.6%) patients reached satisfactory sagittal alignment at the last follow-up. This result demonstrated that using the arthroscopic technique and ACL drill guide could restore the posterior fragment well. Although our study highlighted the importance of anatomic alignment, further investigations are warranted to establish the correlation between radiographic alignment and clinical outcomes. We also used the Rasmussen criteria for further radiographic assessment, and 98.9% of cases achieved good or excellent results. The outcome is comparable to other series using ORIF or other minimally invasive techniques [34–36]. Between the groups, we noticed that the type C1 group was statistically superior to the C3 group. This further revealed the difficulty for the reduction in the C3 group, even if most of our cases in C3 could achieve good to excellent results and only two cases had fair outcomes. Rasmussen clinical assessment was also applied to the series, with all but one of the patients reporting a good or excellent prognosis. In contrast to the radiologic evaluation, there was no significant difference between the three groups. This might also indicate that the advantages of avoiding extensive stripping of soft tissue led to satisfactory outcomes, even if the posterior slope might increase in the long-term follow-up.

The complication rate of complex tibial plateau fractures was high [1, 36–38]. Lee et al. reviewed bicondylar plateau fractures and reported an overall complication rate as high as 39% after operative treatment [1]. Deep wound infections, nonunions and failed treatment requiring revision are common complications due to poor soft tissue conditions combined with high-energy trauma [21, 39]. Ochen et al. retrospectively collected 214 cases with AO/OTA 41-C or Schatzker V/VI tibial plateau fractures and noted that infection occurred in 12% of patients after ORIF, and three-fourths of the infections were deep infections [40]. In a recent systemic review comparing ORIF and ARIF for plateau fractures, the reported complication rates were 5.6% and 9.1% in the ARIF and ORIF groups, respectively. Even if there was no significant difference, the ARIF group showed lower complication rates than the ORIF group [41]. In our series, the complication rate was 6.5%, which was superior to the prognosis reported in complex tibial plateau fractures using ORIF. Four cases of infection were noted, and half of them were deep wound infections and might initially be related to open fractures. The higher incidence in our study is reasonable because most of the fracture patterns using ARIF in previous series were simple plateau fractures. Two cases (2.2%) were converted to TKA due to grade 4 OA. The incidence in the current series is relatively low compared to that in previous studies. Dreumel et al. [42] reported a 7.3% incidence rate in which TKA was eventually required in plateau fractures after 1 year of follow-up on average. Elsoe et al. [2] found that 5.7% of tibial plateaus were treated with a TKA in a large series collected from 5290 patients. Conversely, a superior prognosis for arthroplasty due to OA was reported recently. Ochen et al. [40] reported that the incidence for subsequent arthroplasty following initial ORIF in complex fractures was 3%, which was 6 of 214 cases. Dreumel et al. [42] reported that the rate of arthroplasties was 2.4% in 83 intra-articular tibial plateau fractures. The comparably satisfactory rate in our series might be due to the direct visualization of the joint surface for reduction. We assumed that the minimally invasive soft tissue stripping that led to faster rehabilitation could also be another reason for the low rate of conversion to TKA. Some studies have reported that high-energy complex fracture patterns are contraindications for knee arthroscopy due to the potential risk of iatrogenic compartment syndrome resulting from arthroscopy fluid extravasation [43, 44]. However, in our series, none of the complications were attributed to the arthroscopic procedure. This may be due to the use of temporary external fixation in cases involving open fractures or significant soft tissue swelling at our institution. The ARIF procedure was then performed after a delay of 1 to 2 weeks. To our knowledge, this was the first study to report the long-term prognosis of ARIF in complex tibial plateau fractures. We believe that the use of meticulous arthroscopic techniques for the reduction could achieve excellent results.

Our study has some limitations that should be mentioned. First, this is a retrospective series, and all the limitations of retrospective studies apply to our cohort. Second, plain radiographs were used for evaluation postoperatively rather than CT scans and might cause some bias due to rotation or different positions. Third, surgeries were performed by a single surgeon. The possibility cannot be ruled out that the good prognosis in our study may be due to the practiced skills and training by the experienced surgeon. Fourth, our study lacked a control group of ORIF for comparative analysis. Finally, we also had a moderate rate of loss to follow-up. However, to our knowledge, this is the largest cohort to evaluate long-term outcomes focusing on complex tibial plateau fractures.

## Conclusions

This study demonstrated that complex tibial plateau fractures can be successfully treated using arthroscopy-assisted reduction and minimally invasive internal fixation. Most patients exhibited good to excellent clinical outcomes, low complications and good maintenance of radiography in mid- to long-term follow-up. In our experience, there was a higher incidence of increased slope in type C3 fractures. The posterior fragments thus should be reduced meticulously.

## Data Availability

The datasets analyzed in the current study are available from the corresponding author on reasonable request.
